# Assessing Multiple Factors Affecting Minority Participation in Clinical Trials: Development of the Clinical Trials Participation Barriers Survey

**DOI:** 10.7759/cureus.24424

**Published:** 2022-04-23

**Authors:** Karen Allison, Deepkumar Patel, Ramandeep Kaur

**Affiliations:** 1 Department of Ophthalmology, Mount Sinai Hospital, New York, USA; 2 Public Health, New York Medical College, School of Health Science and Practice, Valhalla, USA; 3 Epidemiology and Public Health, New York Medical College, School of Health Science and Practice, Valhalla, USA

**Keywords:** survey methodology, minority health, diversity and inclusion, health equity, clinical trial

## Abstract

The purpose of this review is to analyze factors that influence individuals' decisions to participate in clinical trials focusing on racial and ethnic disparities that exist in clinical trials. These factors are then used to develop a survey that may be used in a clinical setting to further understand specific factors affecting participation. A comprehensive search of electronic databases was carried out for publications from 2010 to 2021 using Preferred Reporting Items for Systematic Reviews and Meta-Analyses (PRISMA) guidelines. After reviewing the data, the predominant factors that were encountered in the search were then commented upon and reviewed to create an evidence-based questionnaire. ​​​​Using the comprehensive search, factors that affect clinical trial participation were identified. These factors were then used to create a comprehensive, evidence-based questionnaire to be implemented in a clinical setting to conduct and analyze the factors impacting participation in clinical trials. ​​​​​​​Understanding the factors that primarily impact an individual's decisions to participate or not participate in a clinical trial allow researchers to implement changes to decrease the hesitancy regarding participation.

## Introduction and background

Clinical studies refer to research using human participants to add to the available medical knowledge. The two primary types of clinical studies include clinical trials (interventional studies) and observational studies. In clinical trials, participants are given specific interventions, such as drugs or devices, procedures, or modifications in the participants' behavior [1]. Clinical trials provide a "gold standard" for research-generated evidence in healthcare. The success of a clinical trial is dependent upon patient recruitment. Participation in clinical trials allows participants to play an active role in their own health, gain access to new research treatments, increase their options for treatment when the available options have failed, obtain expert medical care at leading health facilities during the trial, and contribute to the advancement of medical knowledge [1].

Despite the critical role of clinical trials in advancing medical innovation, recruiting participants for trials is an arduous task. The recruitment process is extremely time-consuming for the researchers, thereby adding to the cost of the clinical trial. According to one study, "only 55% of trials recruited their originally specified target sample size, 78% of the trials recruited 80% of the original target, and almost one-third of trials received an extension of some kind" [2]. Recruitment of racial and ethnic minorities tends to be even more difficult for researchers.

Currently, African Americans only make up 15% of the minority participants in clinical trials. Likewise, Hispanics make up 7.6% of research participants [3]. A meta-analysis analyzing the prevalence of participants from racial/ethnic minorities groups compared to White individuals in open-angle glaucoma clinical trials reveals that proportions of Black participants did not significantly increase from 1994 to 2019 [4]. Lower numbers of minority participants are attributed to historical events involving minorities. The lower participation may be attributed to the significant human participant abuses in medical research. For example, the Tuskegee Syphilis Study studied the effects of untreated syphilis in African Americans, and the experiment continued even when treatment for syphilis was available. This unethical study, along with others, resulted in African Americans and other minorities being wary of clinical trials. However, it is necessary to analyze and increase minority participation in clinical trials as it is critical for the development and understanding of new therapies [3]. Individuals from different racial and ethnic groups may react differently to medical products. As a result, participation of all, including minorities, is essential in understanding the similarities and differences that may be present in people of different demographic. An increase in minority participants in clinical trials creates the generalizability of the research. Furthermore, minority participation in clinical trials is necessary to create equality and eliminate disparities in the field [3]. Since live subjects are the backbone of clinical trials, successful patient recruitment is a necessary first step for starting and running a clinical trial. 

In 2020, the Federal Drug Administration released a Drug Trials Snapshot providing an overview of demographic characteristics of participants in clinical trials for drugs. As per the analysis, the participation in clinical trials by subpopulation for new molecular entities and therapeutic biologics approved in 2020 was 56% females, 75% Whites, 8% Black African Americans, 6% Asians, 11% Hispanics, 30% age 65 and older, and 54% of the participants were in the United States [5]. The results indicated an increase in female participation in clinical trials. However, the ethnic diversity of participants in clinical trials remained low. It is critical to understand the factors impeding participation in clinical trials as successful trials must represent the population. With the development field of pharmacogenomics, it is essential to have a diverse study group. Thus, patient recruitment and retention are essential for deriving conclusive results from the clinical trial that may reflect the broader population.

Methods 

**Figure 1:**
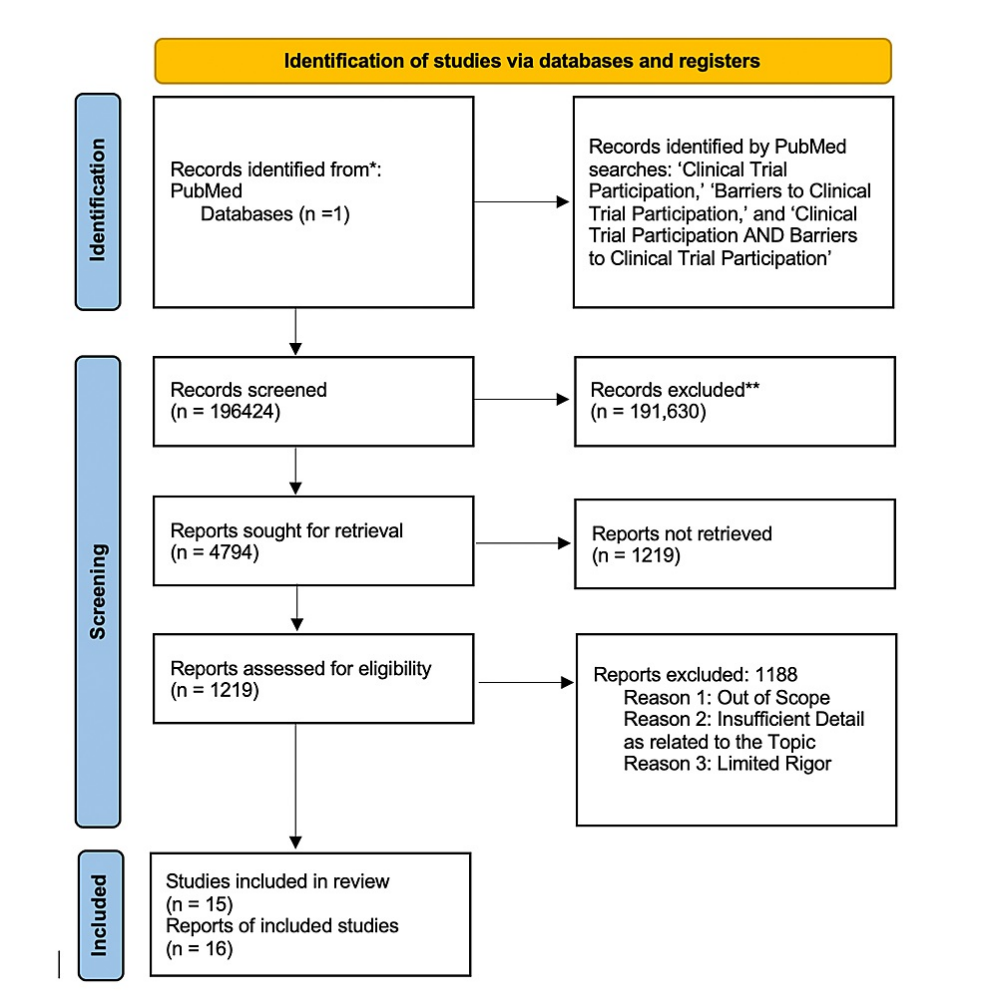
PRISMA flow diagram PRISMA - Preferred Reporting Items for Systematic Reviews and Meta-Analyses

A systematic review using the Preferred Reporting Items for Systematic Reviews and Meta-Analyses (PRISMA) guidelines was undertaken to define factors that affect the decision of individuals to participate in clinical trials (see Figure 1). To identify relevant and up-to-date studies, the literature used to define the factors was published in 2010 or after. Electronic databases, including PubMed and Google Scholar, were searched from 2010 to 2021. 

Keyword terms used included "clinical trial participation", and "barriers to participation in clinical trials". A list of factors impacting participation was created based on the results of the abovementioned searches. The list was then condensed to include twelve factors that affect clinical trial participation. These factors were used to create an evidence-based questionnaire that may be distributed to patients in a clinical setting in order to analyze patient responses regarding what primarily affects their decision to participate in clinical trials. 

## Review

Factors affecting clinical trial participation

There are many factors that can impact one's decision to participate in a clinical trial, ranging from personal interest to affordability. Patients can also be encouraged to participate through incentives, such as money or health benefits, which can be motivating factors encouraging participation. However, this review highlights the barriers to participation. These barriers were chosen because they indicate issues in the healthcare community, particularly issues with clinical trials, that can potentially be improved to enhance the clinical trial experience. Therefore, this review does not include personal interest or disinterest in clinical trials in the discussion. The barriers chosen were also most prominent in the literature. Accordingly, the list of factors affecting clinical trial participation is organized into three categories: psychological and personal, logistical, and healthcare-related reasons. These categories are interrelated due to their close association.

Perceived Personal Risk/Fear 

Given that clinical trials are, in fact, trials, patients are naturally fearful of the unknown impacts of the interventions being tested. In most cases, patients themselves make the final decision regarding clinical trial participation, and they "are most concerned with obtaining the best treatment for their disease" [6]. As a result, one reason why patients decline to participate in clinical trials is fear. Patients report fear regarding two major concerns: "fear of being a "guinea pig" [6] as well as fear of "possible unknown side effects" [7]. Thus, not knowing the (in)effectiveness or potential harms of a trial treatment can lead to patients' decisions to not participate in clinical trials. A recent review of over 400 studies regarding recruitment to medical studies identified fear as one of the most common reasons cited by patients for not participating in clinical trials [7,8]. Yet this topic remains underexplored in recruitment studies [8]. 

Trust in the Medical Community 

Generally speaking, trust in the medical community has been declining in the U.S. over the past few decades. Only 23% of Americans have "a great deal or quite a lot of confidence" in the medical community [9]. This "medical mistrust" results from several factors, such as wasteful healthcare spending and approximately 250,000 annual deaths due to medical errors. Additionally, people are particularly skeptical of medical experimentation due to past ethical breaches by the medical community. People still remember and fear cases such as Henrietta Lacks [10], a woman whose cells were used for research without her knowledge or consent, and thalidomide babies [11], a group of thousands of babies who were born with defects after their mothers were prescribed thalidomide during pregnancy. These examples spur medical mistrust in the general population. 

Additionally, mistrust towards the medical community can also stem from community-based experiences and/or identity-based discrimination against some participants. In particular, African Americans are notably hesitant to participate in clinical trials, and their mistrust is a result of centuries of medical abuse and discrimination. The infamous Tuskegee experiment is an example of such unethical abuses. In this experiment, the participants were not told that the trial intended to study the progression of syphilis. They only received placebos; even after penicillin became the recommended treatment for the disease, they received no effective medical care, and as a result, they died or experienced complications from untreated syphilis [12]. These abuses against the African American community go far beyond this one trial; as recently as the 1990s, esteemed academic institutions carried out blatantly racist and abusive trials using African American boys. Moreover, this mistrust is exacerbated by discrimination and bias in the healthcare system. A recent literature review found that 84% of studies "established evidence of pro-White clinicians or bias toward light-skin/anti-Black, Hispanic, and other minority population" [13]. Moreover, "current occasions of perceived or real racism or discrimination exacerbate mistrust" in the African American community [14]. Hence, medical mistrust remains a prominent barrier to minority participation in clinical trials. 

Ethnic and Cultural Background

Oftentimes, cultural outlooks towards medicine, and especially experimental medicine, can be different. For example, a study compared the attitudes of patients in the U.S., rural China, and urban China regarding clinical research. The study found that while 20-30% of American patients had no concerns about research participation, only 1-10% of Chinese patients shared that attitude. Additionally, personal safety was a much more prominent concern for the Chinese patients (~60%) than for the American patients (~20%), while American patients were more concerned about privacy than their Chinese counterparts. The authors of the study note that these differences could be due to Chinese patients having less prior research participation, having less familiarity or trust in the research and regulatory oversight process, having less trust in physicians and pharmaceuticals due to reports of corruption scandals, and/or being used to China's more relaxed privacy laws [15]. Thus, Chinese patients' concern for safety arises from several cultural and even political factors. 

Similarly, studies in the U.S. have identified participation barriers for different migrant communities. Two separate studies on the South Asian community and the Mexican-American community respectively highlighted similar concerns that prevented participation in clinical trials [16,17]. These concerns included fear of deportation, lack of clinical trials available to these communities, and language barriers (among other common concerns like logistics). Language barriers, in particular, are highly salient reasons for the lack of minority participation in clinical trials, and these barriers can hinder minority participants' understanding of and comfort with the entire clinical research process. Further, these barriers impact communities differently. For example, Spanish translation is more readily available in the U.S. than in any other language; however, due to the diversity of the Hispanic community, there is a difference in the level of understanding of various translated terms (i.e., clinical trials vs. experimental studies) [16]. On the other hand, South Asians are excluded from clinical trials due to language barriers more than "White Europeans or those of other ethnicities" [17]. Therefore, more cultural sensitivity in the clinical trials can increase minority participation.

Relationship with Primary Care Provider

The physician-patient relationship provides a foundation for clinical care and encompasses elements of trust, knowledge, regard, and loyalty [18]. This relationship may have positive and negative implications on clinical care. Three basic models of patient care include the active-passive model, guidance-cooperation model, and mutual participation model. The active-passive model refers to the physician acting upon the patient and is usually ideal for emergency situations. In the guidance-cooperation model, the physician is placed in a position of power due to tier medical knowledge that the patient lacks. The mutual participation model refers to a relationship in which the doctor and patient have an equal partnership [18].

In a guidance-cooperation model and mutual partnership, the physician has the opportunity to create an environment in which the patient is heard and respected. Hence, he or she would be more open to participating in or learning about clinical trials.

Diversity in Healthcare/Primary Investigator

Primary investigators represent key stakeholders in the clinical trial process; therefore, they can be meaningful contributors to increasing the diversity of the clinical trial participants. However, unconscious biases by primary investigators in trials impact the profile (make-up) of clinical trials. Likewise, participants tend to be comfortable with individuals of similar backgrounds. At Stanford University, researchers recruited 1,300 African men [19]. The men were asked to complete a general health survey and then offered a free health screening. Some of the men were seen by Black doctors. At the same time, some of the men were seen by non-Black doctors. Results indicated that men who saw the Black doctors were 56% more likely to get a flu shot, 47% more likely to agree to diabetes screening, and 72% more likely to accept a cholesterol screening [19]. The study mentioned above reflects upon the importance of diversity in healthcare and its impact on creating a safe space for minorities. 

To cater to the minority population, a personalized approach is essential to meet their healthcare needs. Diversity in healthcare and primary investigators eases the ability to collaborate with individuals from diverse cultures while also improving their healthcare experiences and outcomes [20].

Lack of Health Education and Knowledge About Clinical Trials

A primary barrier to enrolling patients for an existing trial is a lack of awareness of clinical trials, especially within minority groups [21]. Many times, patients are unaware of ongoing clinical trials. A study was conducted to evaluate whether providing brief educational material about clinical trials increased the willingness to participate. Participants completed an anonymized electronic survey measuring their impressions of and willingness to enroll in clinical trials. The results indicated that participants were more likely to enroll in a clinical trial after reading the material [22]. This study emphasizes the importance of educating individuals on clinical trials. Awareness through word of mouth by providers, easy-to-understand reading material, or other means of education contributes to an increased likelihood of participation. 

Time Commitment

Many individuals may be reluctant to participate in clinical trials due to the demanding time commitment. Clinical trials usually last at least a few years (depending on the drug or disease being studied). For example, it is reported that the median duration for a clinical trial in non-oncology cases is between 5.9 and 7.2 years, while the median duration of oncology trials is 13.1 years [22]. Additionally, over the past decade, there has been an increase in the duration of trials, a trend that is likely to continue due to the COVID-19 pandemic [23,24]. As a result of the long-lasting nature of clinical trials, participants are often hesitant to make commitments. According to one study, 48% of participants said that time commitment would influence their willingness to participate in trials [25]. While many studies listed time commitment as a potential barrier to patient participation, authors Walsh and Sheridan note that patients often cite time-consuming protocol requirements such as "regular and stringent monitoring of participants", "frequent medical consultations", "time spent within hospitals", "following specific regimens", "having to complete questionnaires", and "keeping a diary log" as time costs that prevent participation in clinical trials [26]. Finally, time is a relative concept, and patient demographic can impact how patients view time commitments for a trial. While younger patients are more concerned about taking time off from work, older patients are concerned about their access to the study drug after their trial participation ends [27].

Costs of Participation

Transportation costs and site location also hinder clinical trial participation due to inaccessibility. Limited access to trial sites can impact the participation ability of certain segments of the population more than others. These include the elderly, especially those who have mobility issues [28], pregnant women [29], rural populations [30], etc. In a 2017 study, researchers reached out to 843 clinical trial sites in the United States. Of the clinical trial sites that responded, 95% of the respondents indicated that transportation efforts would improve recruitment efforts. Furthermore, 63% of the responding clinical trial sites indicated that it would help ensure that participants were recruited on time [31].

In addition to transportation costs, patients are also concerned about other financial costs of participating in a trial, which rank high among the reasons for not participating [6].

Some patients worry about their insurance coverage. While Medicare and many private insurance policies provide coverage for clinical trials, state Medicaid programs provide a varying range of coverage, which means that some patients might not be able to afford clinical trial participation due to a lack of insurance coverage [32]. Finally, the time-consuming nature of clinical trials can also prove costly, especially for lower-income people, who cannot afford to take time off from their work, childcare, and other routine commitments [7,17,26].

Education Level of Participants

The level of educational attainment significantly impacts participation in clinical trials. Lower levels of education tend to attribute to limited medical knowledge. Limited medical knowledge then creates barriers to clinical trial access and helpful resources to deal with certain diseases [32]. Research also indicated that information offered regarding clinical trials tends to be catered toward patients with high educational attainment. A study conducted in 2003 found that consent form failed readability guidelines and included language complexity not suitable for people with low reading levels [33].

A survival analysis study of 6,166 breast cancer patients conducted between 1987 and 2003 had only 12% of participants who did not have a high school diploma. At the same time, 31% of the national population does not have a high school diploma. Thus, the survival analysis study underrepresented patients with lower educational attainment [34].

Socioeconomic Status of Participants

Much like education, socioeconomic status is also linked to participation in clinical trials. Education itself is closely linked to and even considered a valid surrogate for socioeconomic status. On most, if not all, socioeconomic indicators, those with lower status are generally underrepresented in clinical trial participation. Those who participate in trials are usually more likely to be employed and insured, have higher incomes, and have more wealth [33]. The reasons behind the inverse relationship between low socioeconomic status and participation in clinical trials are often combined with the other factors discussed in this paper. In particular, "hidden costs, such as travel to and from clinics, the need for extra childcare, and the loss of income due to missed work will be more significant for those of lower socioeconomic status" [33]. According to one study, patients with lower socioeconomic status were less likely to participate in a clinical trial in both univariate and multivariate models - both yielding statistically significant results [35]. Hence, there is an obvious class disparity in clinical trial participation. 

Ineligible Due to Comorbidities

Comorbidities, the total burden of (chronic) illness other than the specific disease of interest, affect an individual's ability to participate in clinical trials [36]. Often, patients who present with one chronic illness tend to have other coexisting conditions. Furthermore, the likelihood of comorbidities increases with age. In clinical trials, individuals with comorbidities are underrepresented because comorbidities may create potential confounding factors that researchers then have to address [37]. These confounding factors (the comorbidities) impact the reliability of the results. The decision to include or exclude participants to impact the results of the study conflicts with ethical principles in clinical trials. The study by Marrie et al. analyzed multiple sclerosis (MS) and the implications of participation in clinical trials due to physical and psychiatric comorbidities [36]. 

Discussion

To gauge the impact of the above-mentioned factors in a clinical setting, an evidence-based questionnaire was created (see Appendix). Demographics of the respondents were established through questions regarding age, ethnicity, and education level. These demographics are essential to understanding how the demographics of the respondents may affect their responses to the questions. The survey included questions addressing the personal/psychological, logistical, and health-related factors. The goal of the questionnaire is to understand the factors that primarily affect individuals from participating in clinical trials. 

Administering the questionnaire in a clinical setting will allow researchers to use the responses to conduct a statistical analysis. The statistical analysis will present the issues that primarily affect their patients.

## Conclusions

Understanding the results and feedback of the survey is important to ensure patients are given the resources necessary to ensure they understand what clinical trials encompass. Upon analyzing the responses, clinical researchers should assess the results to see which factors primarily affect the population in question. Assessing these factors should allow researchers to understand what changes need to be implemented in order to ensure the participation rate of individuals and make them feel more comfortable with clinical trials.

## Figures and Tables

**Figure 2:**
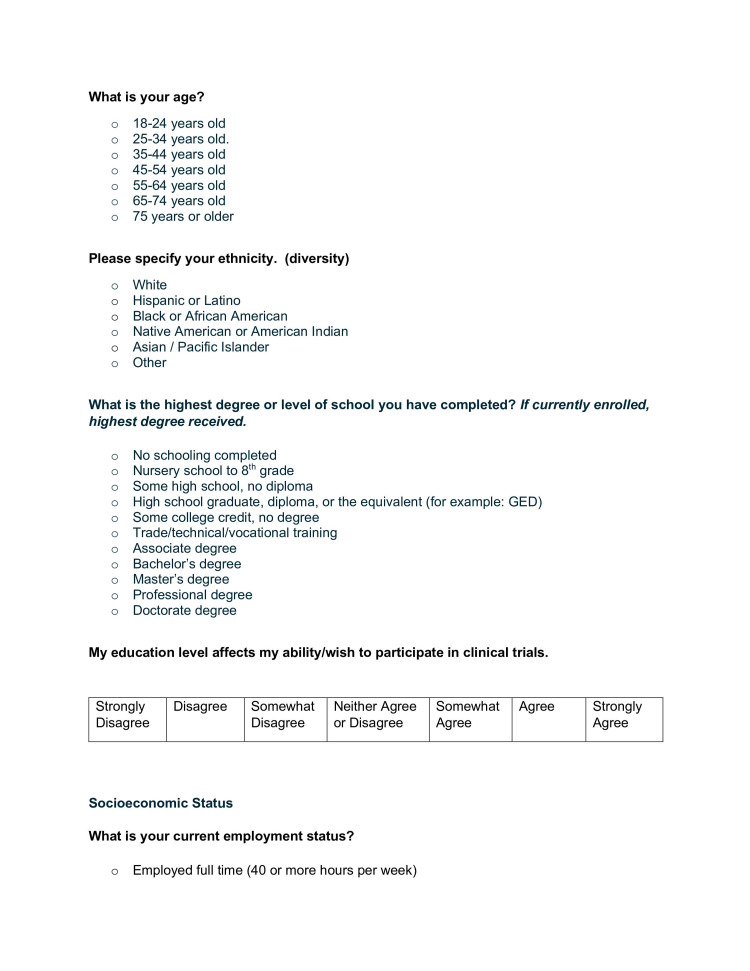
Questionnaire (page 1)

**Figure 3:**
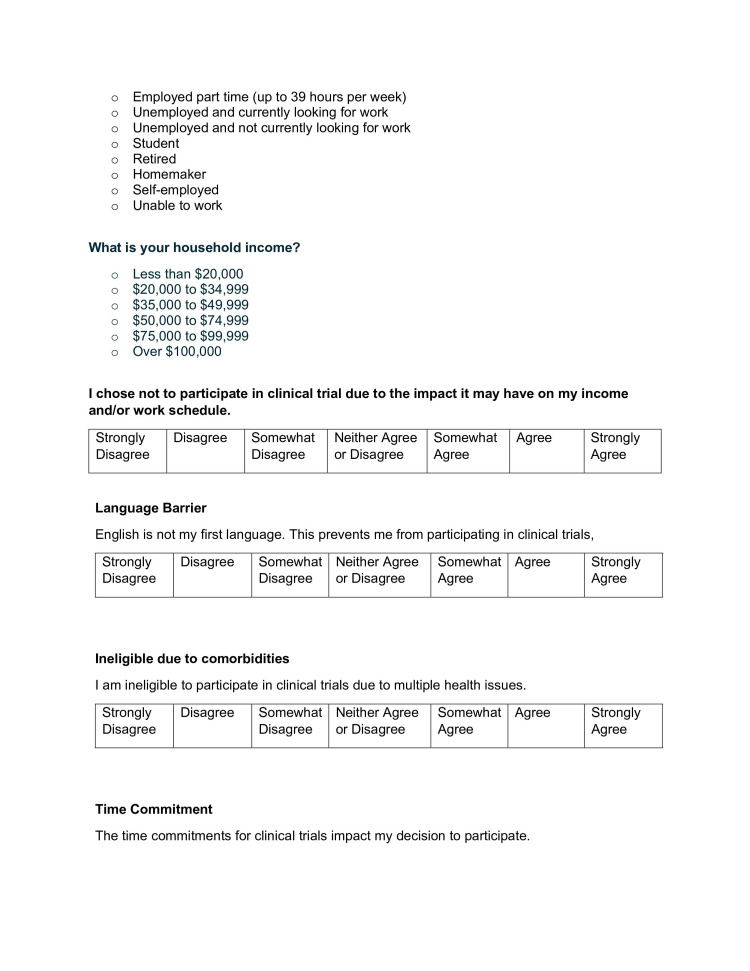
Questionnaire (page 2)

**Figure 4:**
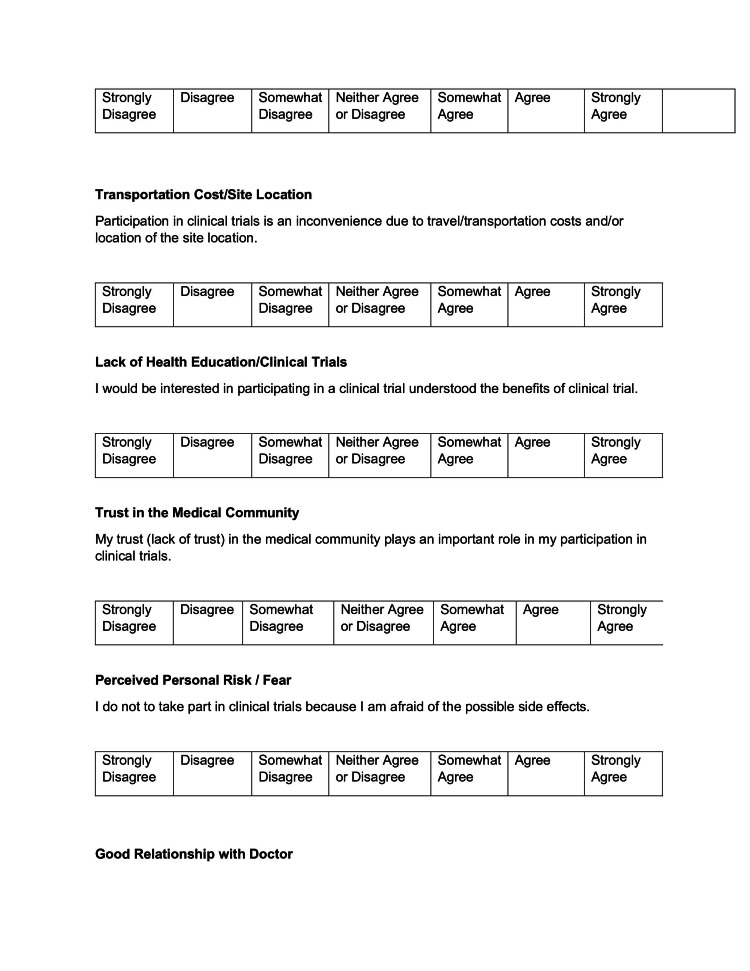
Questionnaire (page 3)

**Figure 5:**
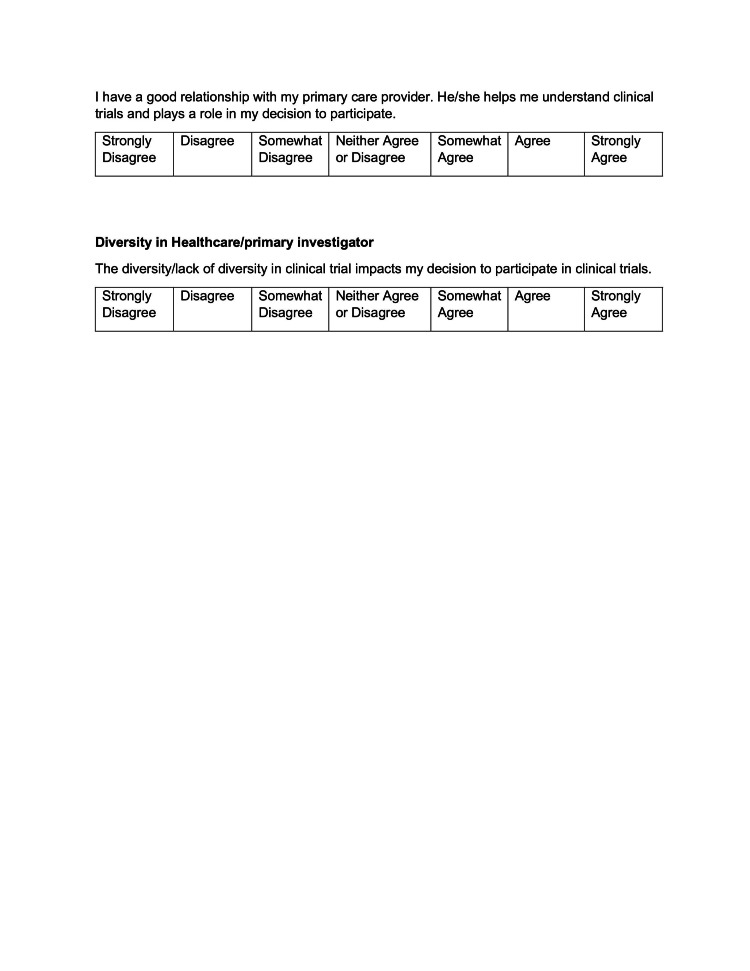
Questionnaire (page 4)
